# Monoclonal antibodies for human and porcine histamine *N*-methyltransferase (HMT) facilitate protein expression and localization studies

**DOI:** 10.1007/s00011-016-0987-1

**Published:** 2016-09-08

**Authors:** Hubert G. Schwelberger, Johannes Feurle, Gunnar Houen

**Affiliations:** 1 Molecular Biology Laboratory, Department of Visceral, Transplant and Thoracic Surgery, Medical University Innsbruck, Schöpfstraße 41, 6020 Innsbruck, Austria; 2 Statens Serum Institut, Artillerivej 5, 2300 Copenhagen, Denmark

**Keywords:** Histamine *N*-methyltransferase, Histamine metabolism, Monoclonal antibodies, Protein expression, Protein localization

## Abstract

**Objective:**

The lack of suitable antibodies for the histamine inactivating enzyme histamine *N*-methyltransferase (HMT) has so far prevented the direct analysis of HMT proteins in man and other mammals.

**Methods:**

A series of monoclonal antibodies was produced by immunizing mice with human and porcine HMT expressed in vitro. Antibodies were characterized by immunoblotting and immunohistochemical staining.

**Results:**

Six different monoclonal antibodies specific for human HMT and four different monoclonal antibodies specific for porcine HMT were obtained that can detect HMT with up to tenfold greater sensitivity than the most sensitive enzymatic assays currently available. Using these antibodies allowed us to confirm the expression and cellular localization of HMT in various human and porcine tissues, where the presence of the enzyme had previously been deduced from activity measurement and HMT mRNA analysis. Immunohistochemical staining of human and porcine tissue sections clearly showed that HMT is a cytosolic protein, which is localized in specific cells of most mammalian tissues.

**Conclusions:**

The new monoclonal antibodies not only allow a comprehensive quantitative evaluation of the expression of HMT at the cellular level in man and other mammals but will also facilitate sensitive analyses of disease-associated alterations of this protein.

## Introduction

Histamine is an important mediator of many biological processes including inflammation, gastric acid secretion, neuromodulation, and regulation of immune function acting through four different G-protein-coupled receptors [1, 2]. Due to its potent pharmacological activity at very low concentrations, the synthesis, transport, storage, release and degradation of histamine have to be carefully regulated to avoid undesirable reactions. Histamine is formed by decarboxylation of the amino acid l-histidine in a reaction catalyzed by the enzyme histidine decarboxylase (HDC, EC 4.1.1.22) [3, 4]. The major routes of histamine inactivation in mammals are oxidative deamination of the primary amino group, catalyzed by diamine oxidase (DAO, EC 1.4.3.22), and methylation of the imidazole ring, catalyzed by histamine *N*-methyltransferase (HMT, EC 2.1.1.8) [4–6].

HMT catalyzes the transfer of a methyl group from S-adenosyl-l-methionine (SAM) to the secondary amino group of the imidazole ring of histamine forming *N* ^*τ*^-methylhistamine [6]. Human HMT is a small monomeric protein of 33 kDa consisting of a single polypeptide chain of 292 amino acid residues. HMT does not appear to carry any modifications and the enzyme does not require any cofactors for its activity. HMT has a two-domain structure with the larger *N*-terminal domain being a classic methyltransferase fold with an SAM binding motif [7]. Human HMT is encoded by a single gene designated HNMT that has six exons and has been mapped to chromosome 2q22.1 [8]. HMT is highly specific for histamine and does not show significant methylation of other substrates. Apart from its reaction products, the enzyme is strongly inhibited by the SH-group reagents *p*-chloromercuribenzoate and *N*-ethylmaleimide and by the antimalarial drugs quinacrine and amodiaquine [6]. HMT appears to be a cytosolic protein that is responsible for the inactivation of intracellular histamine, which is either synthesized in the cell or taken up from the extracellular space after binding to one of its receptors present on the cell surface or by plasma membrane transporters [4].

Histamine *N*-methyltransferase is detectable in most tissues of mammals by activity measurements or mRNA analysis experiments [9–11] but the expression and cellular localization of the protein have not been determined for most tissues especially in man, which is mainly due to the lack of antibodies specific for HMT. Recently, we succeeded in making antibodies for porcine and human DAO, the enzyme catalyzing the alternative route of histamine inactivation, and these turned out to be invaluable tools for the study of this enzyme [12, 13]. To close the gaps in our knowledge of HMT expression, localization and function, we therefore, set out to produce highly sensitive and specific monoclonal antibodies also for the human and porcine HMT proteins.

## Materials and methods

### Preparation of recombinant HMT proteins

Full-length human and porcine HMT cDNAs [9, 10, 14] were amplified by PCR with specific primers from total human and porcine kidney cDNA, respectively, and cloned in frame into the bacterial expression vector pGEX-2T (GE Healthcare, Vienna, Austria). Each recombinant plasmid was transformed into the protease-deficient strain *E. coli* BL21 to produce glutathione S-transferase (GST) fusion proteins according to manufacturer’s instructions (GE Healthcare, Vienna, Austria). Briefly, recombinant bacteria were grown at 37 °C with slight agitation (100 rpm) in 500 ml YTA (16 g/l tryptone, 10 g/l yeast extract, 5 g/l NaCl, 100 mg/l ampicillin, pH 7.0) to an OD_600 nm_ of 0.5 and fusion protein expression was induced for 4 h by addition of 0.1 mM isopropyl-β-d-thiogalactopyranoside (IPTG, Roche, Vienna, Austria). Bacteria were harvested by centrifugation for 5 min at 4000*g*, 4 °C, washed with cold deionized water, and lysed in lysis buffer (20 mM bis·Tris·HCl, pH 7.0, 5 mM dithiothreitol) containing Complete Protease Inhibitor Cocktail (Roche, Vienna, Germany) using a French Press at 600 psi. Both constructs were expressed at high levels and produced largely soluble fusion proteins that were recovered from the supernatants of bacterial lysates after centrifugation for 5 min at 5000*g*, 4 °C and purified to near homogeneity by chromatography on glutathione sepharose (GSTrap FF, GE Healthcare, Vienna, Austria) according to manufacturer’s instructions. The purified GST fusion proteins, which had a similar HMT enzymatic activity as the enzyme prepared from kidney tissue [15] were dialyzed against PBS buffer (10 mM NaH_2_PO_4_, pH 7.2, 150 mM NaCl) and used for immunizations.

### Generation of HMT specific antibodies

**Fig. 1:**
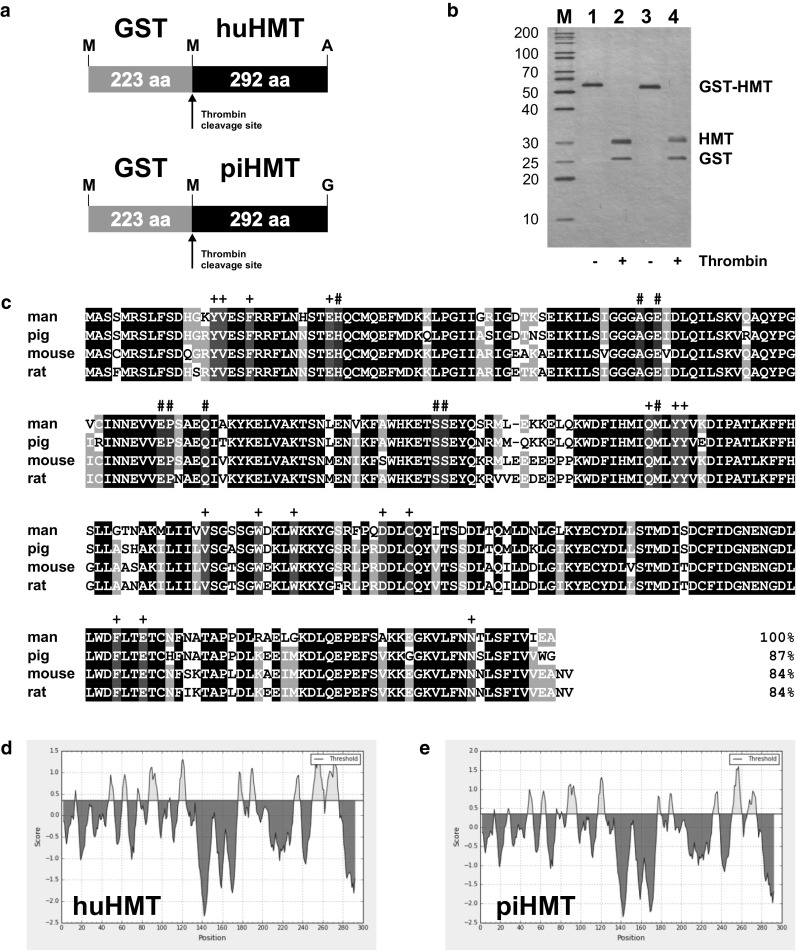
Recombinant HMT antigens used for immunizations. **a** Recombinant human and porcine GST-HMT fusion proteins cloned in the bacterial expression vector pGEX-2T. **b** 12.5 % Silver-stained polyacrylamide gel of the purified human (*lane 1*) and porcine (*lane 3*) GST-HMT fusion proteins used for the immunization of mice and the HMT and GST products resulting from cleavage with thrombin protease (*lanes 2* and *4*). The sizes of molecular weight markers (*M*) are given on the left in kilodalton. **c** Sequence alignment and percent sequence identity of the HMT proteins from man, pig, mouse and rat obtained with the NCBI Constrained-based Multiple Alignment Tool (www.ncbi.nlm.nih.gov/tools/cobalt/cobalt.cgi?link_loc=BlastHomeLink). Residues identical in all four proteins are shaded *black*, residues identical in three proteins are shaded *gray*, and residues that have been shown to interact with histamine and S-adenosylhomocysteine in human HMT [7] are shaded *red* (indicated by *plus symbol* on top) and *blue* (indicated by *hash* on top), respectively. Antigenicity plots of human (**d**) and porcine (**e**) HMT produced with the BepiPred Linear Epitope Prediction Tool (www.tools.immuneepitope.org/bcell) [32] show similar predicted B-cell epitopes (*yellow* peaks on top of the threshold line with reference to amino acid positions) for both protein sequences

Balb/c mice (*n* = 2–4) were immunized i.p. four to five times with recombinant antigens GST-huHMT and GST-piHMT, respectively, (Fig. 1) mixed with alhydrogel (9.8 mg/ml). Each mouse received 20 µg antigen + 1 mg Al(OH)_3_ per injection. After the second and the following injections serum samples were collected and tested by ELISA for the presence of antibodies. When the titer had increased to a level above 1:10,000, selected mice received an i.p. injection of 20 µg antigen in physiological saline (150 mM NaCl). After 4 days, the mice were sacrificed and the spleens removed. Homogenization and disintegration of the spleens, fusion and cloning followed standard procedures essentially as described earlier [16], except that the use of fibroblast feeder cells was substituted with HybER™ medium (SSI, Copenhagen, Denmark). Animal experiments were carried out in accordance with the European Communities Council Directive of 24 November 1986 (86/609/EEC) and were approved by local authorities.

For testing antibody titers by ELISA, antigens (0.5 µg/ml) were coated in Maxisorp plates (Nunc, Denmark) using 100 µl 50 mM sodium carbonate buffer, pH 9.6 per well. All subsequent blocking (5 min), washing (3 × 5 min) and incubation (60 min) steps were done in TTN buffer (25 mM Tris, pH 7.5, 0.5 % Tween 20, 150 mM NaCl). After blocking, wells were incubated 60 min with sera (starting dilution 1:200 and 2× titrations) or culture supernatants and after washing, wells were incubated 60 min with alkaline phosphatase-conjugated goat IgG against mouse IgG (Sigma, St. Louis, USA). Finally, wells were incubated with para-nitrophenyl phosphate (1 mg/ml) in 1 M diethanolamine, pH 9.8 and the absorbance read at 405 nm with background subtraction at 690 nm. The titer of a serum was defined as the dilution giving half maximal absorbance.

### Characterization of HMT specific antibodies

Selected antibody clones (hybridoma culture supernatants or affinity purified immunoglobulins) were further tested for binding specificity and sensitivity using filter strips of human and porcine tissue homogenates. Tissue homogenates were prepared using the AllPrep DNA/RNA/Protein Mini Kit (Qiagen, Hilden, Germany) and dissolving the total precipitated protein in BUD (20 mM bis.Tris.HCl, pH 7.0, 8 M urea, 50 mM dithiothreitol). The protein was diluted with SDS sample buffer and 100 µg was separated on a 10 % SDS polyacrylamide gel [17] and blotted onto a polyvinylidene fluoride (PVDF) membrane [18]. After washing in TBST (50 mM Tris.HCl, pH 7.5, 150 mM NaCl, 0.1 % Tween 20) and blocking non-specific binding sites by incubation for 60 min at 4 °C in TBSTM (TBST containing 2 % non-fat dry milk) the membrane was cut into 20 vertical filter strips each containing circa 5 µg of protein. Each filter strip was incubated for 2–16 h at 4 °C with different dilutions of the monoclonal antibodies in TBSTM, washed 4 × 5 min with TBST, incubated 60 min at 4 °C with horseradish peroxidase-conjugated anti-mouse immunoglobulins (Dako, Glostrup, Denmark) diluted 1:1500 in TBSTM, washed 4 x 5 min with TBST, incubated 5 min with ECL or ECL Prime reagent (GE Healthcare, Vienna, Austria), and exposed to Cronex 5 film (Agfa, Mortsel, Belgium).

For analyses of HMT in human tissues and body fluids, we used homogenates of the macroscopically healthy part of surgical specimens not needed for histopathological evaluation as well as blood and urine samples from healthy volunteers (*N* = 10, 5 male, age 23–49). Tissue samples were immediately frozen and stored at −25 °C until analyzed. Tissue samples (1–5 mg) were homogenized in 10 volumes of 20 mM bis·Tris·HCl, pH 7.0 containing 10 mM dithiothreitol and Complete Protease Inhibitor Cocktail (Roche, Vienna, Austria) for 5 min at 30 Hz using a TissueLyser II homogenizer (Qiagen, Hilden, Germany). The homogenate was cleared by centrifugation for 10 min at 20,000*g*, 4 °C and the supernatant containing the total soluble protein was stored at −25 °C until analyzed. Blood samples drawn from the antecubital vein were allowed to clot for at least 2 h at 4 °C, centrifuged for 10 min at 1500*g*, 4 °C and the clear serum was stored at −25 °C until analyzed. Urine samples were cooled on ice, centrifuged for 10 min at 20,000*g*, 4 °C and the clear supernatant was stored at −25 °C until analyzed. Protein concentration of all samples was determined by the Bradford method [19] using a commercial kit (Biorad, Vienna, Austria). Proteins in different samples were characterized by SDS polyacrylamide gel electrophoresis (SDS-PAGE) [17] or by two-dimensional isoelectric focusing (IEF)/SDS-PAGE [20] and the presence of HMT was analyzed by Western blotting onto PVDF membranes essentially as described for the filter strips above, using the mouse monoclonal antibodies at optimum concentrations. HMT activity was determined by transmethylation of histamine with S-adenosyl-l-[methyl-^14^C]methionine (GE Healthcare, Vienna, Austria) as described previously [21] and the specific activity was calculated in microunits per milligram protein where 1 µU converts 1 pmol of substrate per minute at 37 °C. All human samples were obtained for two separate studies approved by the Ethical Committee of the Medical University Innsbruck and carried out in compliance with the Declarations of Helsinki and Istanbul and were analyzed here anonymized without access to patient data.

### Immunohistochemical staining

For immunohistochemical staining, tissues were fixed for 16–24 h in 4 % paraformaldehyde and embedded in paraffin wax. Sections of 5 µm were cut, mounted on silanized glass slides (Menzel, Braunschweig, Germany), and dried 16 h at 50 °C. Slides were dewaxed 4 × 3 min in xylol, 1 × 3 min each in 100 % ethanol, 96 % ethanol, and 80 % ethanol, rinsed for 5 min with water, and autoclaved for 10 min at 121 °C in 10 mM sodium citrate pH 6.0 for antigen retrieval. Slides were washed 2× with TBS (50 mM Tris.HCl, pH 7.5, 150 mM NaCl) and mounted on Coverplates in Coverplate Racks (Thermo Fisher Scientific, Vienna, Austria) for subsequent incubations. Each incubation step was followed by three washes with TNT (TBS containing 0.05 % Tween 20). Endogenous peroxidase activity was blocked by incubation in 1 % H_2_O_2_ for 15 min, endogenous biotin was blocked employing the Biotin Blocking System (Dako, Glostrup, Denmark), and non-specific protein binding sites were blocked by incubation in TNB (TBS containing 0.5 % Blocking Reagent, PerkinElmer, Rodgau, Germany) for 30 min. Slides were incubated for 16 h at 4 °C with the mouse monoclonal antibodies for human or porcine HMT diluted 1:100–1:1500 in TNB and then for 2 h a 25 °C with horseradish peroxidase-conjugated anti-mouse immunoglobulins (Dako, Glostrup, Denmark) diluted 1:100 in TNB. The Tyramide Signal Amplification System (PerkinElmer, Rodgau, Germany) was used according to manufacturer’s instructions for signal amplification. For staining of immunocomplexes, slides were incubated for 5 min with DAB substrate (0.05 % 3,3′-diaminobenzidine, 0.01 % H_2_O_2_, 50 mM Tris·HCl, pH 7.6) and counterstained with Mayer’s hemalum (Merck, Darmstadt, Germany). Slides were dehydrated by incubation for 3 min each in 70 % ethanol, 80 % ethanol, 96 % ethanol, 2× 100 % ethanol, and 2× xylol and coverslips were mounted with Entellan (Merck, Darmstadt, Germany).

## Results

**Fig. 2:**
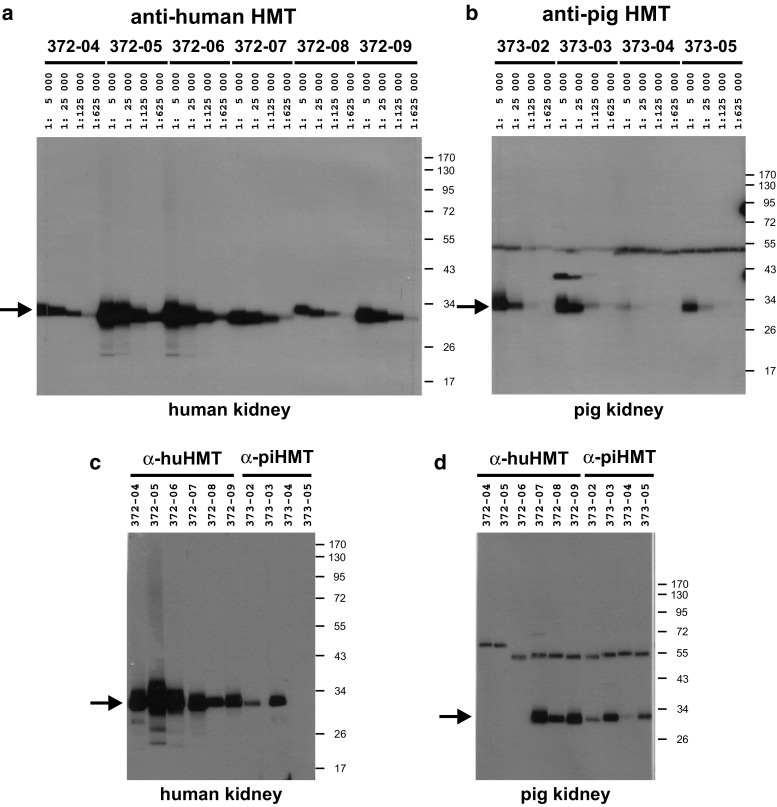
Test of antibody specificity, sensitivity, and cross-reactivity. Filter strips containing approximately 5 µg human kidney homogenate (**a**, **c**) or 5 µg pig kidney homogenate (**b**, **d**) separated on a 10 % SDS polyacrylamide gel were incubated with the indicated dilutions of the mouse monoclonal antibodies (1 mg/ml) for human HMT (HYB372-04/-05/-06/-07/-08/-09) (**a**) or for pig HMT (HYB373-02/-03/-04/-05) (**b**) in TBSTM. **c**, **d** Filter strips were incubated with the indicated antibodies at a dilution of 1:10,000 in TBSTM. Filter strips were then incubated with horseradish peroxidase-conjugated anti-mouse immunoglobulins (1:1500 in TBSTM), followed by ECL substrate, and exposure to film for 1 min (**a**) or 5 min (**b**, **c**, **d**). Sizes of molecular weight markers (*M*) are given in kilodalton on the right and the migration position of HMT at 33 kDa is indicated by *arrows* on the left. The band at ca. 55 kDa visible in all lanes of (**b**) and (**d**) results from a weak cross-reaction of the anti-mouse immunoglobulins with the large fragment of porcine IgG present in the samples. The exact migration position of the visible bands varies slightly in different lanes because filter strips from different individual blots were used for this experiment

Using GST fusion proteins of recombinant human and porcine HMT expressed in bacteria as antigens for immunizations of mice (Fig. 1), six clones were selected that produced monoclonal antibodies with high binding specificity and affinity for human HMT (HYB372-04/-05/-06/-07/-08/-09) and four clones for porcine HMT (HYB373-02/-03/-04/-05). The selected antibodies strongly bound to the respective recombinant HMT protein obtained by thrombin cleavage of the GST fusion proteins used for immunization (results not shown). Initial testing of these monoclonal antibodies using human and porcine kidney homogenates showed that all antibodies produced a single strong band at 33 kDa and could be diluted up to 1:625,000 (Fig. 2a, b). Further testing of optimum antibody dilutions using filter strips of human and porcine kidney homogenates gave suitable titers of 1:1000–1:10,000 for different antibodies. Apart from HYB373-03 that showed a weak reaction also with a ca. 40 kDa protein, none of the monoclonal antibodies gave any additional bands when tested with the kidney homogenates. Analysis of antibody cross-reactivity showed that antibodies HYB373-02/-03 resulting from immunizations with porcine HMT also reacted with a 33 kDa protein in human kidney homogenates (Fig. 2c) and that antibodies HYB372-07/-08/-09 resulting from immunizations with human HMT also reacted with a 33 kDa protein in porcine kidney homogenates (Fig. 2d). The relative band intensities observed with identical amounts of loaded protein and identical antibody concentrations indicated that the cross-reacting antibodies bound to the same protein in human and porcine kidney lysates.

**Fig. 3:**
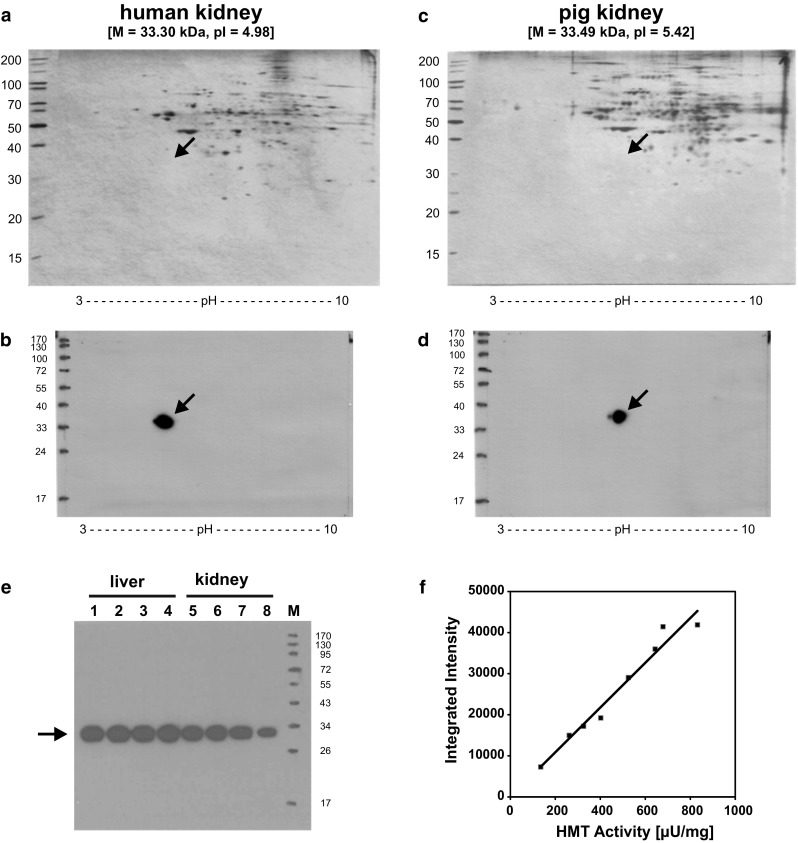
Detection of HMT on 2D-IEF/SDS-PAGE gels and correlation of band intensity with HMT activity. Ten microgram each of a human kidney homogenate (**a**, **b**) or a porcine kidney homogenate (**c**, **d**) was separated by isoelectric focusing on a pH 3–10 gradient IEF gel followed by size separation on a 10 % SDS polyacrylamide gel. Gels were either silver-stained (**a**, **c**) or blotted onto PVDF membranes (**b**, **d**). For detection of HMT, blots were incubated with HYB372-07 (1:6000 in TBSTM) and horseradish peroxidase-conjugated anti-mouse immunoglobulins (1:1500 in TBSTM), followed by ECL prime substrate, and exposure to film for 1 min. The molecular mass and *pI* values calculated for the human and porcine HMT proteins are given in square brackets on top of the gels. **e** 2 µg each of four different human liver homogenates (*lanes 1*–*4*) and four different human kidney homogenates (*lanes 5*–*8*) were separated on a 10 % SDS polyacrylamide gel, blotted onto PVDF membrane, and HMT was detected by incubation with HYB372-05 (1:6000 in TBSTM) and horseradish peroxidase-conjugated anti-mouse immunoglobulins (1:1500 in TBSTM), followed by ECL prime substrate, and exposure to film for 1 min. **f** Integrated intensity determined for the bands on the blot in (**e**) using ImageJ was plotted against the HMT activity in the respective sample. A Pearson’s correlation coefficient of 0.983 with *p* < 0.01 was obtained for this correlation using SPSS Statistics 19 (SPSS Inc., Chicago, USA). Sizes of molecular weight markers (*M*) are given in kilodalton and the migration position of HMT at ca. 33 kDa is indicated by *arrows*

When probing total proteins of human and porcine kidney lysates after separation by two-dimensional IEF/SDS-PAGE with one of the cross-reacting antibodies HYB372-07, a single spot was detected that migrated at ca. 33 kDa at a pH of ca. 5.0 for human kidney (Fig. 3b) and a pH of ca. 5.5 for porcine kidney (Fig. 3d), which is in excellent agreement with the molecular mass and *pI* values calculated for the human and porcine HMT polypeptide sequences, respectively. However, no spots were visible at the respective positions of parallel Silver-stained gels (Fig. 3a, c), indicating the low abundance of the protein in kidney lysates. When blots of liver and kidney homogenates from different individuals were analyzed for the presence of HMT using HYB372-05, the same sharply focused band at 33 kDa was obtained in each case (Fig. 3e), and the intensity of the band was proportional to the HMT enzymatic activity in the respective sample (Fig. 3f), confirming that the antibodies indeed bind to HMT. Even on very long exposures, no significant other bands were visible on the blot showing the absolute specificity of the antibodies. With the most sensitive enzymatic assay currently available [21] it is possible to detect approximately 15 pg HMT. Using the blotting technique with the monoclonal antibodies described here we were able to reliably detect 1.5 pg HMT protein in a dilution series of human liver and kidney homogenates (results not shown), which translates into a ca. tenfold higher sensitivity compared with the activity determination. This excellent sensitivity allows to exclude the presence of significant amounts of HMT in samples where HMT is not detectable by immunoblotting.

Histamine *N*-methyltransferase could be detected on blots of all total human and porcine tissue homogenates analyzed (results not shown), which is in accordance with previous studies analyzing HMT enzymatic activity and HMT mRNA in various porcine tissues [11]. In agreement with HMT activity measurements, HMT could not be detected on blots of total human and porcine blood plasma proteins. Although HMT activity is below the limits of detection in human urine, minute and individually variable amounts of HMT were detectable on blots of all urine samples analyzed.

**Fig. 4:**
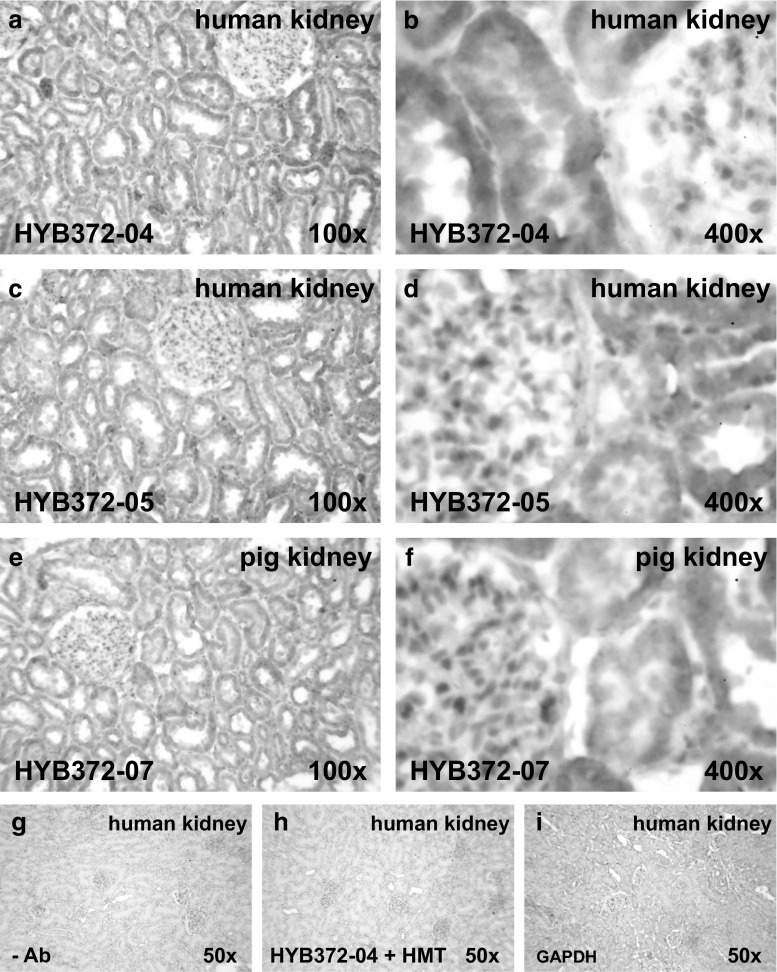
Immunohistochemical staining of kidney sections. Thin sections of human kidney (**a**, **b**, **c**, **d**) and pig kidney (**e**, **f**) were incubated with HYB372-04 (**a**, **b**, diluted 1:1000 in TNB), HYB372-05 (**c**, **d**, diluted 1:1000 in TNB), and HYB372-07 (**e**, **f**, diluted 1:500 in TNB), respectively. Following incubation with horseradish peroxidase-conjugated secondary antibodies and tyramide signal amplification, slides were developed with 3,3′-diaminobenzidine (*brown* staining of immunocomplexes) and counterstained with Mayer’s hemalum (*blue* staining of nuclei and cell membranes). Control incubations omitting the primary antibody (**g**) or pre-incubating the primary antibody with a 25-fold molar excess of the respective recombinant antigen used for immunization (**h**) produced no staining. Staining of parallel sections with an antibody specific for the cytosolic marker glyceraldehyde-3-phosphate dehydrogenase (GAPDH) produced a very similar staining pattern as the HMT-specific antibodies (**i**) except for the fact that GAPDH stains all cells, whereas HMT staining is restricted to specific cells. Slides were photographed at 100× (**a**, **c**, **e**), 400× (**b**, **d**, **f**), and 50× (**g**, **h**, **i**) enlargement

We next tested whether the monoclonal antibodies could detect HMT in tissue sections. When performing immunohistochemical staining on human and porcine kidney sections, identical staining patterns were obtained with all antibodies at dilutions of 1:100–1:1500 as shown exemplary for HYB372-04, HYB372-05, and HYB372-07 in Fig. 4. In both human and porcine kidney, there was prominent staining of tubular epithelial cells (Fig. 4a, c, e) and at higher magnification the staining was diffusely spread over the whole cell as would be expected for an evenly distributed cytosolic protein (Fig. 4b, d, f). Besides, there was significant staining of a few isolated cells that appeared to be monocytes or macrophages. Although human erythrocytes have been described to possess HMT activity [22] there was no clear staining of these cells, which might be due to the low abundance of the protein in erythrocytes. To confirm this assumption, we prepared human erythrocyte lysates and were able to detect minute amounts of HMT on blots and measure a very low HMT activity. Compared with other cells, the relative abundance of HMT in erythrocytes was found to be much lower and apparently not sufficient for detection by the less sensitive technique of immunohistochemical staining.

A comprehensive study investigating the expression and cellular distribution of HMT in various human and porcine tissues is under way and will be presented in a separate communication. The results available so far confirm earlier analyses of HMT activity measurement and HMT mRNA expression in porcine tissues [11] showing the presence of the enzyme in most tissues with a distinct cellular localization. In accordance with the results of immunohistochemical localization of HMT in guinea pig tissues [23], human and porcine HMT appeared to be moderately expressed in epithelial cells of the gastrointestinal tract, the urinary tract, the airway system, the skin, and in hepatocytes. Besides, in most tissues analyzed so far, HMT staining was prominent in a subset of hematopoietic cells of the monocyte-macrophage and dendritic cell lineages. From immunohistochemical staining, the overall HMT tissue levels represented by specific enzymatic activity could be deduced from the expression level in individual cells and the abundance of HMT expressing cells in the respective tissue. Noteworthy, immunohistochemical staining worked well also on sections from tissues processed for routine histopathological evaluation by fixation in 10 % formaldehyde and embedding in paraffin wax.

**Fig. 5:**
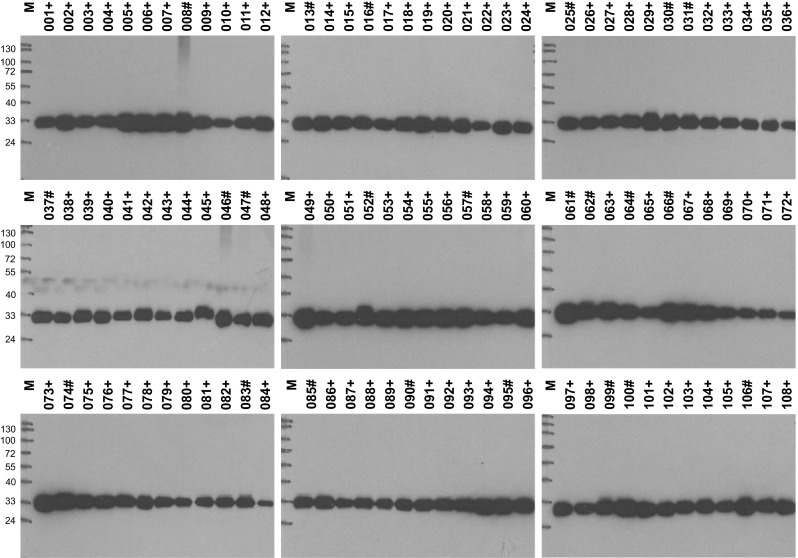
Analysis of HMT in human transplant tissue. Aliquots of tissue samples from 108 consecutive control biopsies of human kidney (*green numbers marked with plus sign*, *n* = 85) and liver (*blue numbers marked with hash*, *n* = 23) grafts obtained for routine diagnostic procedures were homogenized and cleared by centrifugation. For each sample, 2 µg total protein was separated on a 10 % SDS polyacrylamide gel, blotted onto a PVDF membrane and HMT was detected with HYB372-07 (1:6000 in TBSTM) and horseradish peroxidase-conjugated anti-mouse immunoglobulins (1:1500 in TBSTM), followed by ECL Prime substrate, and exposure to film for 1 min. Sizes of molecular weight markers (*M*) are given in kilodalton on the *left*. The exact migration position of HMT at ca. 33 kDa varies slightly in different lanes due to the different salt concentration in individual samples

To test the utility of the monoclonal antibodies for investigations of HMT in human samples, we analyzed the presence of HMT in a series of consecutive control biopsies of kidney and liver transplant tissue obtained for routine diagnostic procedures (Fig. 5). Using blots of homogenates prepared from minute amounts of tissue not used for histological assessment, HMT was clearly detectable in just 2 µg of total protein loaded and showed considerable variation of abundance in different kidney and liver grafts. Although HMT could easily be quantitated from these blots by scanning the band intensities, exact quantification in a routine setting would certainly warrant development of a simpler and faster ELISA test. All HMT monoclonal antibodies should work fine in an ELISA because this was the procedure used for original screening of the antibody clones. Further studies are in progress to clarify the variations in HMT levels in kidney and liver grafts and to assess if this parameter might be diagnostically useful. In any case, this study demonstrates the feasibility of this type of analysis in extremely small samples making use of the excellent sensitivity of the new HMT antibodies.

## Discussion

A series of monoclonal antibodies exhibiting a high specificity and sensitivity for human and porcine HMT, respectively, was obtained by immunizing mice with HMT proteins expressed in vitro. All antibodies recognized a single protein band of 33 kDa corresponding to monomeric HMT on immunoblots of tissue homogenates and except for HYB373-03 did not bind to any other human or porcine protein in the samples analyzed. With these antibodies it was possible to detect HMT with tenfold higher sensitivity than with the most sensitive radioenzymatic assay in use [21]. Furthermore, these antibodies work excellent in immunohistochemical staining of human and porcine tissue sections and thus facilitate the sensitive and specific detection of the HMT protein at the cellular and subcellular level.

The specificity of the antibodies was demonstrated by detection of a single band of the expected size of 33 kDa on immunoblots of different human and porcine samples (Fig. 2a, b), correlation of the intensity of this band with HMT activity in the respective sample (Fig. 3e, f), and detection of a single spot of expected size and pI value on blots of two-dimensional gels of human and porcine kidney homogenates, respectively (Fig. 3b, d). Although not presented here, we also performed immunoprecipitation experiments that showed that HYB272-04/-05/-06/-08/-09 and HYB373-03 but not HYB372-07 and HYB373-02/-04/-05 precipitated HMT from human and porcine kidney and liver lysates prepared under non-denaturing conditions, indicating that these antibodies also bind to the native HMT protein. As was evident from antibody dilution experiments, different antibodies exhibited outstanding but different sensitivities for the detection of HMT on immunoblots of tissue homogenates (Fig. 2a, b) and in immunohistochemical staining. Furthermore, HYB372-04/-05/-06 bound only human HMT, HYB373-04/-05 bound only porcine HMT, whereas HYB372-07/-08/-09 and HYB373-02/-03 bound both human and porcine HMT (Fig. 2c, d). Therefore, our collection contains both antibodies recognizing species-specific epitopes but also common epitopes and these might be useful for analyses of HMT in other species.

All antibodies detected HMT by immunohistochemical staining of tissue sections and produced identical staining patterns when used at optimum concentration (Fig. 4). In all tissues analyzed so far, we observed relatively uniform staining of specific cells as would be expected for a soluble cytosolic protein. Although a cytosolic localisation of HMT could be deduced from the facts that mammalian HMT polypeptide sequences (Fig. 1c) lack sorting signals for other cellular compartments and that HMT activity is usually associated with the soluble fraction obtained by differential centrifugation of cell lysates [15, 24, 25], we here provide direct immunohistochemical evidence for the subcellular localisation of the protein in man and pig. Our findings are in accordance with results of HMT localisation experiments in human astrocytes reported by Yoshikawa and colleagues [26]. As will be reported elsewhere, HMT was found to be present in epithelial cells of the gastrointestinal tract, the urinary tract, the airway system, the skin, and in hepatocytes, which agrees well with immunohistochemical analysis of HMT expression in guinea pigs [23]. Additionally, prominent HMT staining was found in human and porcine hematopoietic cells that morphologically appeared to be macrophages and dendritic cells but this has to be confirmed by staining with cell-specific markers. We did not see HMT staining of blood vessels or neuronal cells as has been reported in the bovine nervous system [27] and it is not clear if this is due to species differences or antibody specificity. We also did not see HMT staining of erythrocytes, where the sensitivity of immunohistochemistry appears not to be sufficient to detect the low levels of the enzyme present in these cells [22]. Given the cytosolic localisation of HMT, it will be interesting to analyze if HMT expressing cells also express histamine receptors [1, 2] and/or transporters that could mediate histamine uptake [28–30] to clarify the physiological role of the enzyme in histamine inactivation.

We also included a small exploratory study analyzing HMT in minute amounts of samples from control biopsies of kidney and liver grafts (Fig. 5) to demonstrate that the antibodies described here can be used in a routine setting. It is apparent from the blots that the amount of HMT protein varies significantly in different samples, where we carefully checked protein loading and protein patterns of parallel silver-stained gels and verified uniform HMT staining of the tissue sections, from which the samples for protein analysis had been obtained. Detailed evaluation of these differences in HMT tissue levels and correlation with organ quality and function is certainly worthwhile especially for the kidney, where the selection of non-invasive biomarkers for injury and function is very limited [31]. As kidney injury is usually associated with changes in the urine, it is interesting to note that both histamine inactivating enzymes HMT and DAO can be detected in human urine with the antibodies described here and earlier [13] although the enzymatic activities are beyond the limits of detection. If found to be diagnostically useful, a routine ELISA test for HMT determination in human urine could easily be set up with these antibodies.

In conclusion, the ten monoclonal HMT antibodies described here represent excellent tools for the sensitive and specific detection of the human and porcine HMT proteins allowing a comprehensive evaluation of the expression and cellular localization of the enzymes in man and pig. Furthermore, these antibodies will facilitate a better understanding of the role of HMT in histamine inactivation in different cell types as well as sensitive analyses of disease-associated alterations of the enzyme.

## Abbreviations

DAO: Diamine oxidase
GAPDH: Glyceraldehyde-3-phosphate dehydrogenase
GST: Glutathione S-transferase
HDC: Histidine decarboxylase
HMT: Histamine *N*-methyltransferase
IPTG: Isopropyl-β-d-thiogalactopyranoside
SAM: S-adenosyl-l-methionine
